# A generator-matrix causal-inference framework separates measurable aging biomarkers from mortality-driving latent dynamics in humans

**DOI:** 10.64898/2026.07.05.26356402

**Published:** 2026-07-09

**Authors:** Masato Tanigawa, Takafumi Iwaki

**Affiliations:** 1 Department of Biophysics, Faculty of Medicine, Oita University, Yufu, Oita, Japan

**Keywords:** ageing, epigenetic clock, Mendelian randomization, colocalization, reprogramming, reliability theory, biomarkers of aging, causal inference

## Abstract

A central challenge in computational geroscience is to distinguish molecular quantities that *predict* mortality from those that *causally drive* it. Epigenetic clocks and aging biomarkers are increasingly used as if they were that mechanism, yet this is rarely tested directly. This distinction also bears on competing theories of aging: damage/reliability (A), hyperfunction/mTOR–IIS (B-1), and information loss (B-2). Although individual aging proteins have been tested piecemeal, no study has asked, in one framework, what fraction of mortality is measurable, whether it is causal, and whether it is reversible. Using only public, de-identified data, we evaluate this three ways. First, a Markov **generator-matrix** model of hallmark-load dynamics with death as an absorbing state, fitted by Bayesian inference through a joint biomarker-and-mortality likelihood to NHANES with linked mortality (n=23,844) and replicated in the Health and Retirement Study (HRS), decomposes Gompertz acceleration into *visible* (measured-biomarker-driven) and *latent* components. Second, a positive-control-calibrated, two-platform cis-pQTL Mendelian-randomization and colocalization design (UKB-PPP, deCODE) against parental-lifespan GWAS tests whether the latent’s measurable components are causal. Third, a clock battery (Horvath, chronological; DamAge, causality-enriched damage) tests reversibility in cellular reprogramming. Within the model, ~92% of Gompertz acceleration is assigned to a latent component not captured by measured blood-biomarker axes (NHANES 92.5%, HRS 91.6%); the latent is partly encoded in DNA-methylation signatures but not transcription. The known causal proteins are detected (LPA p=9×10^−12^; IL6R p=2.8×10^−5^), yet the latent’s components, across inflammatory, renal and growth-signalling (IGFBP3, IGF-1) axes, are null and do not colocalize on either platform. Reprogramming reverses the chronological clock (−11 to −22 yr) but not the causality-enriched damage clock. The model-inferred mortality-driving component is largely latent to accessible biomarkers; its measurable molecular proxies show no supported causal effect where the design detects known causes; and the causality-enriched damage-clock signal is resistant to partial reprogramming.

## Author summary

Aging “clocks” and blood biomarkers are increasingly used as if they measured the process that makes us age and die, yet a thermometer reflects a fever without causing it. We asked whether the molecular quantities we can measure are the *cause* of mortality or merely downstream *markers*, and built a computational framework that tests this in three independent ways. First, a dynamical reliability model that treats death as an absorbing state, fitted to two large population datasets, indicated that roughly 92% of how fast mortality rises with age is not captured by the blood biomarkers we can measure. Second, a genetic causal test (a natural experiment using inherited gene variants), calibrated with proteins already known to affect lifespan, showed that the measurable markers, across inflammation, growth signalling and the epigenetic clock, do not causally change human lifespan, whereas the known-causal proteins do. Third, cellular “rejuvenation” reversed a chronological clock but not a causality-enriched damage clock. Collectively, these results indicate that popular aging measures track mortality *risk* rather than its *cause*, and we provide a reusable computational framework for telling the two apart.

## Introduction

The last decade produced a proliferation of “aging clocks” and circulating biomarkers of biological age, now entering clinical trials and geroprotector evaluation as putative readouts of the aging process. Their use rests on an implicit premise: that the molecular quantity being measured is, or closely tracks, the *mechanism* that makes us age and die. A thermometer reflects fever but cooling the thermometer does not cure the disease; whether aging biomarkers are mechanism or thermometer is rarely tested directly.

The question is sharpened by three influential and partly competing theories of what drives aging mortality (for a critique of the hallmarks paradigm, see [1]). (A) **Damage accumulation / reliability theory** holds that aging is the macroscopic form of stochastic somatic damage approaching a tolerance threshold; the damage is real and largely irreversible. (B-1) The **hyperfunction** view holds that aging is a quasi-programmed continuation of nutrient/growth (mTOR–insulin/IGF-1) signalling rather than damage [2]. (B-2) The **information theory** of aging holds that aging is reversible loss of epigenetic information, with partial reprogramming restoring youthful states [3,4]. These theories make different predictions about whether the mortality-driving component is measurable, causal, and reversible.

The empirical state of the field leaves the question open. Epigenetic clocks (Horvath [5], Hannum [6], PhenoAge [7], GrimAge [8], DunedinPACE [9]) and proteomic age predictors consistently *predict* mortality and morbidity, and second-generation, mortality-trained clocks do so most strongly; yet prediction is not causation, and the molecular features a clock weights need not lie on the causal path to death. Genetic causal tests of individual aging-associated proteins have been performed piecemeal and give mixed or null results, but rarely within a single framework that calibrates power with positive controls or guards against linkage-disequilibrium confounding by colocalization. In parallel, partial reprogramming reverses epigenetic clocks in vitro and improves some tissues in vivo [4,10,11], fuelling claims that aging is reversible; but whether reprogramming reverses the layer that actually drives mortality, as opposed to a cosmetic chronological tag, has not been separated using clocks that distinguish chronological age from causal damage. What is missing is a test that asks, in one coherent design, three questions at once: how much of mortality acceleration is even captured by accessible measures, whether those measures are causal, and whether they are reversible.

We approach this as a problem in **evaluation science** rather than mechanism discovery: instead of proposing a new aging mechanism, we ask whether the quantities we can already measure are the causal, reversible drivers of mortality, and we adjudicate A / B-1 / B-2 with three orthogonal lines of evidence. First, we quantify how much of mortality acceleration is even *captured* by accessible biomarkers, using a dynamical reliability model (a Markov **generator matrix** with death as an absorbing state) grounded in cross-species reliability theory and the mortality-rate-doubling-time–lifespan scaling recorded across species in AnAge [12,13,14]. Second, we test whether the measurable molecular components are **causal** for human lifespan using cis-pQTL Mendelian randomization plus colocalization on two independent proteomic platforms, anchored by known-causal positive controls so that a null cannot be dismissed as low power. Third, we test **reversibility** by asking whether cellular reprogramming reverses a chronological clock and a causality-enriched damage clock equally.

We find that the three lines converge: the model-inferred mortality-driving component is largely latent to accessible measures, its measurable molecular proxies are non-causal across the inflammatory, growth-signalling and epigenetic axes, and the causality-enriched damage layer resists reprogramming. The popular clocks encode mortality risk, not mechanism.

### Contribution.

Individual elements have precedent: phase-type mortality models with biological-aging states [15,16]; piecemeal causal nulls for inflammatory markers [17]; causality-enriched clocks [18]; the broader distinction between the predictive utility of aging clocks and their mechanistic interpretation, recently discussed in perspective [19]; and standardized frameworks for benchmarking the predictive performance of aging biomarkers [20]. The contribution here is not that argument or predictive benchmarking, but its integrated empirical adjudication of cause: a single computational framework that subjects the marker-versus-cause question to three orthogonal, convergent tests: (i) a **generator-matrix reliability model** whose joint biomarker-and-mortality likelihood decomposes mortality acceleration into visible biomarker-driven and latent components; (ii) a **positive-control-calibrated, two-platform cis-pQTL MR and colocalization layer** that tests whether the measurable components are causal; and (iii) a **clock-battery reversibility test** that separates chronological-clock reversal from damage-clock reversal. The specific new causal nulls (renal and growth-signalling axes on parental lifespan), the resolution of the partial-reprogramming window, and the hierarchical reading of A / B-1 / B-2 are developed in the Discussion.

## Results

### 2.1 A generator-matrix model: ~92% of mortality acceleration is latent to measurable biomarkers

**Figure 1.**
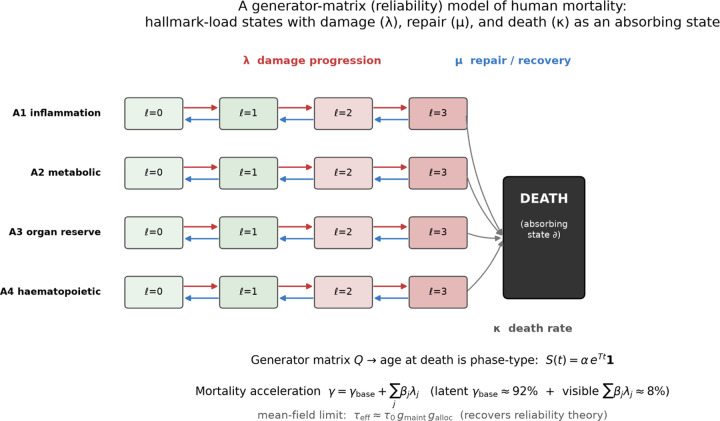
A generator-matrix (reliability) model of human mortality. Hallmark-load states on four axes (A1 inflammation, A2 metabolic, A3 organ reserve, A4 haematopoietic) progress under damage rates λ and recede under repair rates μ; death is an absorbing state entered at rate κ. The population age-at-death distribution is the phase-type first-passage time S(t)=αe^{Tt}1; the Gompertz mortality acceleration γ decomposes into a latent baseline (~92%) and a visible part driven by measured-axis dynamics (~8%), and the mean-field limit recovers the reliability decomposition τ_eff≈τ0·g_maint·g_alloc. (Schematic; source fig_concept.py.)

We modelled the organism as occupying hallmark-load states (the accumulated burden on each aging-hallmark axis) [21] whose transitions are governed by a Markov generator matrix Q, with death an absorbing state, so that the population distribution of age at death is a phase-type first-passage time. (Fig. 1) Hallmark-load axes were constructed from the nine PhenoAge NHANES biomarkers, kept multi-dimensional as four axes (inflammation, metabolic, organ/renal, haematopoietic) rather than collapsed to a single index. Using ergodicity (the death-age distribution of many individuals constrains the latent transition dynamics), we jointly estimated the state dynamics (per-axis drift *λ*_*j*_) and the proportional-hazard mortality contribution (*β*_*j*_) in a single Bayesian model, with the baseline Gompertz rate (under the Gompertz law, mortality rises near-exponentially with age) given a cross-species τ-informed prior.

Decomposing the posterior Gompertz acceleration γ into a **visible** part driven by measured-axis dynamics (∑_*j*_ *β*_*j*_ *λ*_*j*_) and a **latent** baseline (*γ*_base_), the latent fraction was **92.5% [92.0–93.0]** (Table 1; S1 Fig) (NHANES 1999–2010 pooled, n=23,844, deaths=5,005; all $\hat{\text{r}}=1.000$). The same decomposition in an independent cohort and period (HRS, with future mortality) gave **91.6% [89.6–93.4]** (S2 Fig). Within this model, the measured blood hallmark axes account for only ~7–8% of Gompertz mortality acceleration, with ~92% assigned to a latent component not captured by these accessible measures. Among the four measured axes, the haematopoietic and metabolic axes were the largest contributors to the small visible acceleration (per-axis *β* · *λ* = 0.0032 and 0.0020 yr^−1^), yet their summed contribution remained roughly an order of magnitude below the latent baseline; the decomposition gave a sharply peaked posterior and was stable to the number of axes and load levels (Methods).

### 2.2 The latent is partially encoded in DNA-methylation systemic signatures

To localize the latent, we added mechanism-mapped DNA-methylation surrogate modules (protein-trained, hence less circular than mortality-trained composite clocks) to the four blood axes in HRS, one at a time. The latent shrank most with a **renal (DNAm cystatin C) module (−26.5 pt)**, then **inflammaging (DNAm CRP+GDF15+B2M, −20)** and **senescence (DNAm PAI-1+TIMP1, −17)**, while a **metabolic module did nothing (0)**. Generic chronological epigenetic age (Horvath) added nothing beyond blood (mortality HR 1.03, NS); a mortality-trained composite (GrimAge) captured ~2/3 of the latent but is intrinsically circular. Whole-blood **transcription** of the same inflammaging/senescence genes did not capture the latent (mortality HR ≈ 1.0). The latent therefore behaves as a slowly accumulating, DNAm-integrated systemic load (inflammaging/senescence/renal), not a transcriptional snapshot, and is invisible to generic chronological clocks (S3 Fig).

### 2.3 The latent’s measurable components are not causal for lifespan: two-platform MR + colocalization

If these molecular markers drove mortality, genetically-predicted higher levels should shorten lifespan. We tested this with cis-pQTL MR plus colocalization against parental-lifespan GWAS [22], on **two independent platforms** (UKB-PPP Olink; deCODE SomaScan), with known-causal **positive controls** to calibrate power.

**Figure 2.**
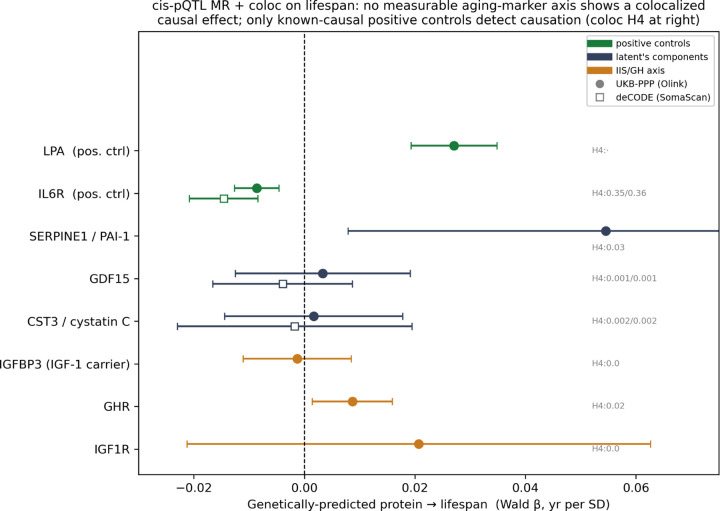
cis-pQTL Mendelian randomization and colocalization of measurable aging-marker axes on human lifespan. Forest plot of genetically-predicted protein effect on parental lifespan (Wald β, yr per SD; bars = 95% CI) for known-causal positive controls (green: LPA, IL6R), the latent’s inflammatory/senescence/renal components (dark: SERPINE1, GDF15, CST3/cystatin C), and the growth-signalling/IIS axis (orange: IGFBP3, GHR, IGF1R), on two proteomic platforms (circles UKB-PPP Olink; open squares deCODE SomaScan). Right-hand annotations: colocalization PP.H4 (shared causal variant). Positive controls (LPA, IL6R) have CIs excluding zero; every measurable molecular component is either null (CI crosses zero) or, where nominally non-zero (SERPINE1, GHR), fails to colocalize with lifespan (PP.H4≈0). (Source: MR_ukbppp.csv, MR_COLOC_decode.csv, MR_COLOC_iis.csv, COLOC_ukbppp.csv.)

Positive controls behaved as expected: **LPA** (β=+0.027 yr/SD, p=9×10^−12^) and **IL6R** (p=2.8×10^−5^ [UKB-PPP], p=3.8×10^−6^ [deCODE]) showed clear causal effects, confirming the pipeline detects causation at these instrument strengths and outcome sample sizes. Instruments were strong where proteins could be instrumented (F = 1499–20031); the positive-control effects were precise (LPA β = +0.027 [95% CI 0.019, 0.035] yr/SD; IL6R β = −0.0086 [−0.013, −0.0047]), whereas the latent components’ confidence intervals spanned zero (cystatin C β = +0.0017 [−0.014, 0.018]; GDF15 +0.0033 [−0.013, 0.019]). Results were unchanged across the two MR/colocalization implementations and the three longevity outcomes tested. In stark contrast, the latent’s measurable components were **null even with stronger instruments**: GDF15 (F=1924) p=0.68; **cystatin C / CST3** (F=1499) p=0.84, replicated on deCODE (GDF15 p=0.54; CST3 p=0.87). Moreover, colocalization reinforced this: cystatin C carried an extremely strong cis-pQTL (PP.H1 = 0.97 [UKB-PPP], 0.98 [deCODE]) yet shared **no** causal variant with lifespan (PP.H4 ≈ 0), i.e. its levels are tightly genetically controlled but causally disconnected from longevity. The single nominal signal (SERPINE1, p=0.022) did not colocalize (PP.H4 = 0.03) and is attributable to LD. B2M and IL6 had no usable cis-pQTL on **either** platform (a biological, not platform-specific, limitation), and TIMP1’s instrument lies on chromosome X, absent from the autosomal lifespan GWAS (Fig. 2; Table 2). The conclusion, that the measurable inflammatory/senescence/renal proxies are markers not causes, is stable across platform, instrument strength, and LD confounding.

### 2.4 The growth-signalling (hyperfunction) axis is also non-causal

Because hyperfunction (B-1) locates the driver in nutrient/growth (mTOR–IIS) signalling, we tested the MR-tractable proxy of that axis, circulating IGF-axis and GH proteins. The IGF-1 carrier **IGFBP3** carried a very strong cis-pQTL (F=837) yet was **null** for lifespan with **no** colocalization (PP.H1 = 0.98, PP.H4 = 0), the same signature as cystatin C. **IGF-1 itself** (deCODE) likewise failed to colocalize with lifespan (PP.H4 = 0.01), backing up the IGFBP3 null with the direct marker. IGFBP1 and IGF1R were null; GHR’s nominal signal (p=0.018) did not colocalize (PP.H4 = 0.02). Thus the measurable circulating growth-signalling axis joins the inflammatory/renal axis as a marker, not a cause, of human longevity (Fig. 2; Table 2). We emphasise that circulating pQTL-MR indexes the IIS axis but cannot measure intracellular mTOR activity; this does not refute the hyperfunction mechanism but shows it has no causal handle in the measurable circulating proxy, the same latency wall seen for A and B-2.

### 2.5 Reprogramming reverses the chronological clock but not the causality-enriched damage layer

Finally we tested reversibility (the core prediction of B-2). To separate a “cosmetic” chronological layer from a mortality-relevant damage layer, we applied a chronological clock (Horvath) and a **causality-enriched, tissue-independent damage clock, DamAge** (derived by Mendelian randomization of CpGs) to cellular reprogramming datasets.

**Figure 3.**
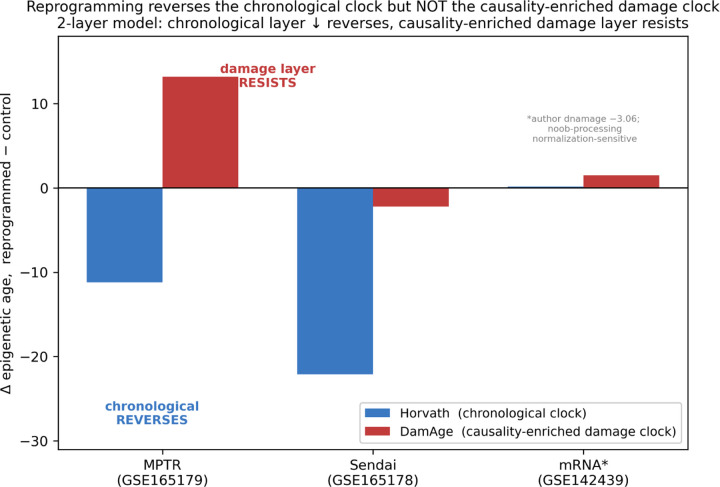
Reprogramming reverses the chronological clock but not the causality-enriched damage clock. Δ epigenetic age (reprogrammed − control) for a chronological clock (Horvath, blue) and a causality-enriched damage clock (DamAge, red) across reprogramming datasets (MPTR GSE165179; Sendai GSE165178; mRNA GSE142439). Horvath reverses strongly; DamAge resists. (Source: REV_damage.csv, REV_GSE165178.csv, REV_GSE142439.csv.)

In transient reprogramming of human fibroblasts (MPTR [23]), Horvath reversed strongly (−11.2 yr) while DamAge did **not** reverse (+13.2). In an independent reprogramming method with an internal control (Sendai, SSEA4^+^ reprogramming vs CD13^+^ non-reprogrammed cells; same study), Horvath reversed even more (−22.1 yr) while DamAge resisted (−2.2 overall; +16.6 during partial reprogramming at day 9, falling only near full iPSC induction at day 15, when somatic identity is lost). Thus the chronological layer reverses consistently across two reprogramming methods, while the causality-enriched damage layer resists during the partial reprogramming that retains cell identity (Fig. 3; Table 3), thereby resolving the chronological-versus-damage dissociation of Ying et al. [18] to the partial, identity-retaining window, where the damage layer resists and falls only as full iPSC identity loss is approached (consistent with the strong DamAge reversal Ying et al. reported at the iPSC endpoint). (In a third, independent-group dataset (Sarkar et al. [24], mRNA reprogramming), the reversal was small and normalization-sensitive; our pipeline reproduced the authors’ DNAmAge at r=0.97, but this dataset is an underpowered test of the dissociation; the strongest two-layer evidence rests on the Gill datasets. An in-vivo mouse partial-reprogramming dataset showed only a small, non-significant chronological reversal after age adjustment.)

## Discussion

Three orthogonal lines of evidence converge on one conclusion. The model-inferred mortality-driving component of human aging is (i) **largely latent** to accessible biomarkers (within the generator-matrix decomposition, ~92%), (ii) **not** the measurable molecular markers (across the inflammatory/renal, growth-signalling, and epigenetic axes, no measurable proxy shows a supported causal effect while known-causal positive controls are detected), and (iii) a **reprogramming-resistant causality-enriched damage layer**.

### A two-layer model.

Reprogramming, and by extension the popular discourse of “rejuvenation,” moves a **reversible chronological tag** (Horvath; cosmetic, not mortality-driving) but leaves a **resistant causality-enriched damage layer** (DamAge / the latent; mortality-driving) intact during the partial reprogramming compatible with retained cell identity. This dissociation reframes what aging clocks measure: the popular clocks are best read as **encoders of mortality risk** (or chronological tags) rather than as measures of the causal mechanism, consistent with the observation that mortality-trained composites capture the latent (circularly) while generic chronological clocks do not.

### Adjudicating A / B-1 / B-2.

The reversible information layer (B-2) demonstrably exists, but it is the *non-lethal* chronological layer; the layer that drives mortality is damage-like and resistant, favouring the damage/reliability base (A). Furthermore, the hyperfunction axis (B-1) has, in our hands, **no causal handle in its measurable circulating proxy**; like A’s damage and B-2’s reversible tag, its mechanism (if any) is not visible in accessible measures. The unifying theme is therefore not a contest between molecular theories but a **hierarchy**: a damage/reliability base that drives mortality but is latent to current measures, beneath a reversible, non-lethal molecular surface (chronological tags, circulating growth-signalling proxies) that is what assays and reprogramming actually move.

### Comparison with prior evidence.

The individual nulls are each consistent with scattered prior reports, which our framework unifies. The cystatin C null accords with its status as a renal-filtration marker rather than a causal longevity factor; circulating CRP has repeatedly failed Mendelian-randomization tests for the outcomes it predicts, and a recent Mendelian-randomization study independently found CRP and GDF15 non-causal for all-cause mortality while IL6 and IL6R were causal [17]; and human IGF-1/insulin–IGF-signalling MR has been inconsistent despite the strong model-organism phenotypes, which act on intracellular signalling that secreted-protein instruments do not capture. Rather than treating these as isolated negative findings, the positive-control-calibrated, two-platform, colocalization-backed design shows them to be a single regularity: measurable molecular age markers, across inflammatory, renal and growth-signalling axes, sit off the causal path to death. That the same class of methods (proteome-wide cis-pQTL MR with colocalization) can recover causal protein effects on human longevity-related outcomes for other proteins [25] indicates that the uniform null across these aging-marker axes reflects their position off the causal path rather than low power, and our positive-control-calibrated design extends the recent all-cause-mortality nulls to parental lifespan and to the renal (cystatin C) and growth-signalling axes. The reversibility result builds on Ying and colleagues’ causality-enriched clocks [18], which uncoupled damage from adaptation and showed DamAge reversing at full iPSC reprogramming: in two independent reprogramming methods we find that within the partial, identity-retaining window the causality-enriched damage layer instead resists, reversing only as full iPSC identity loss is approached, and we tie this resistant layer to the within-human latent. Finally, the picture is compatible with the view that aging clocks track accumulating stochastic variation read out as a clock [26]: a quantity that predicts mortality, can be partially reset, and yet is not the mechanistic driver. Consistent with this, recent Mendelian-randomization work found no evidence that commonly used epigenetic clocks or telomere length causally increased lifespan [27]; the present analysis extends that conclusion from composite clocks to specific protein axes with two-platform colocalization and positive-control calibration, and integrates it with the latent-fraction and reversibility tests. What the latent damage is (accumulated tissue injury, genomic instability, or a senescent-cell burden poorly represented in circulating blood) remains the central open question.

### Implications.

(1) For biomarker-guided geroscience: a measured aging clock or protein shifting under an intervention does not establish that the intervention touched the mortality-driving component; causal/colocalization and reversibility tests of the kind used here should gate such claims. (2) For reprogramming: the chronological reversal that headlines “rejuvenation” is, on these data, the non-lethal layer; demonstrating reversal of the causality-enriched damage layer in vivo (with retained somatic identity) remains the key unmet test. (3) For mechanism discovery: the next task is to **co-develop measures that capture the damage substrate itself**, beginning from the DNAm inflammaging/senescence/renal signatures that partially index it, at tissue and single-cell resolution. More broadly, the framework is reusable: any candidate aging biomarker or geroprotector readout can be passed through the same three gates (latent-fraction accounting, positive-control-calibrated causal MR with colocalization, and chronological-versus-damage reversibility) before it is treated as mechanism. Applying such gates would temper over-interpretation of clock movement in intervention trials and would redirect target discovery from the abundant, measurable, non-causal surface toward the latent damage substrate itself.

## Limitations

The visible/latent decomposition is model-dependent (four linear axes + proportional Gompertz; replicated across two cohorts but not assumption-free), and the identity of the latent (which unmeasured damage) remains unknown. MR is constrained by instrument availability: B2M and IL6 lack usable cis-pQTL on both platforms, TIMP1 and IGFBP2 fall in outcome-GWAS gaps (chromosome X; a chr2 coverage gap), and coloc.abf assumes a single causal variant per region (limiting interpretation at allelically heterogeneous loci such as LPA). Circulating pQTL-MR indexes secreted proteins, not intracellular signalling (mTOR), so B-1 is engaged at its measurable proxy, not its mechanism. Reversibility evidence is predominantly in vitro; the independent-group replication was small and normalization-sensitive, and no mouse causality-enriched damage clock exists to test the damage layer in vivo. DamAge is derived from blood EWAS (pan-tissue but not strictly tissue-independent). HRS follow-up is short; mortality-trained clocks are intrinsically circular. All conclusions concern public deidentified data and are framed in the project’s ethics stance (social-determinant-first interpretation; no genetic determinism).

## Materials and methods

### Unified inference framework

All three analyses share a single Bayesian causal/graphical-inference framework: the generator-matrix model is Bayesian inference over a continuous-time probabilistic graphical (Markov) model with an absorbing state; Mendelian randomization is causal-directed-acyclic-graph instrument design (variant → exposure → outcome); and colocalization (coloc.abf) is Bayesian model comparison over causal configurations (H0–H4). The marker-versus-cause question is thereby treated as one of Bayesian causal inference.

### Data and cohorts

#### NHANES + Linked Mortality.

We pooled the U.S. National Health and Nutrition Examination Survey continuous cycles 1999–2010 [28] (n = 23,844 adults aged ≥ 20 with the nine PhenoAge clinical biomarkers and Public-Use Linked Mortality Files follow-up to 2019; deaths = 5,005). Biomarkers were standardized within cycle to remove assay drift. **HRS.** Replication used the Health and Retirement Study [29] (RAND HRS Longitudinal mortality × Venous Blood Study biomarkers × HRS DNA-methylation clocks), linked by respondent identifier and analysed locally in aggregate (cell sizes ≥ 5) under the HRS data-use terms; analytic n = 3,985, deaths = 725 over a mean 5.7-year follow-up. **GEO** reprogramming methylation: GSE165179 (MPTR), GSE165178 (Sendai), GSE142439 (mRNA/ERA), GSE247179 (chemical), GSE190665 (in-vivo mouse 4F [10]). **pQTL:** UKB-PPP Olink (Synapse syn51365303; [30]) and deCODE SomaScan [31]. **Outcome GWAS:** parental lifespan (GWAS Catalog [32] GCST006697, harmonised GRCh38; [22]); parental top-decile longevity (GCST006698) and parental extreme longevity (GCST003395), and the longevity meta-analysis of Deelen et al. [33], were used as secondary outcomes where available. **Cross-species:** AnAge [13].

### Generator-matrix model of mortality acceleration

We represent the organism as occupying hallmark-load states *s* = (*h*_1_,…,*h*_*m*_) with *h*_*j*_ ∈ {0,…,*L*} the discretized load on axis *j*, plus an absorbing death state *∂*. The nine PhenoAge NHANES biomarkers were grouped, without collapsing to one dimension, into *m* = 4 hallmark axes: A1 inflammation/immune (CRP, white-cell count, lymphocyte %), A2 metabolic/nutrient-sensing (fasting glucose, albumin), A3 organ reserve (creatinine, alkaline phosphatase), A4 haematopoietic (mean cell volume, red-cell distribution width). State transitions follow a continuous-time Markov generator *Q* with damage-progression rates *λ*_*j*_(*ℓ*; **x**), repair rates *μ*_*j*_(*ℓ*; **x**), and an absorbing death rate *κ*(*s*; **x**). Writing the transient block *T* and exit vector **t**^0^ = *κ*, the age at death of an adult started at *a*_0_ from initial distribution *α* is **phase-type**:

$$S(t)=\alpha e^{Tt}1,\;f(t)=\alpha e^{Tt}t^{0},\;\mu (t)=f(t)/S(t).$$

Ergodicity makes the population distribution of age at death informative for *T* without following individuals longitudinally; the role of unobserved frailty heterogeneity in population mortality is classical [34]. Phase-type and inhomogeneous-Markov representations of human mortality, including the interpretation of states as stages of biological aging, are well established [15,16]; those models are fit to mortality data alone, where the intensity matrix is only weakly identifiable, and regressing individual transition rates onto aging markers was noted there as a desirable but overparameterized extension. The present contribution is to break that identifiability with a joint likelihood that ties the stages to measured biomarker axes, and to use the fitted model to decompose Gompertz acceleration into biomarker-driven *visible* and *latent* components. Rates are modulated log-linearly by covariates **x** (sex as the primary stratifier; quantitative exposures, namely smoking pack-years and BMI, as rate covariates; never race or nationality), $\lambda_{j}(\ell ;x)=\lambda_{j}^{0}(\ell )e^{\beta_{j}^{\lambda }\cdot x}$.

For estimation and the visible/latent decomposition we used the joint formulation implemented in bayes_unified_generator.py: a per-axis drift *λ*_*j*_ shared between (i) a state-dynamics likelihood, in which the cross-sectional age gradient of each standardized axis *z*_*ij*_ ~ 𝒩^(^*b*_*j*_ + *λ*_*j*_(*a*_*i*_ – *a*_*c*_), *σ*_*j*_) identifies *λ*_*j*_, and (ii) a proportional-hazard Gompertz mortality likelihood [35] in which accumulating damage raises the hazard, so that the same *λ*_*j*_ contributes a **visible** acceleration ∑_*j*_ *β*_*j*_ *λ*_*j*_ on top of a **latent** baseline *γ*_base_. The total Gompertz acceleration is *γ* = *γ*_base_ + ∑_*j*_ *β*_*j*_ *λ*_*j*_ and the latent fraction is *γ*_base_/*γ*. Priors: *β*_*j*_ ~ 𝒩(0, 0.5), *λ*_*j*_ ~ 𝒩(0, 0.1), *σ*_*j*_ ~ HalfNormal(1), log *λ*_0_ ~ 𝒩(log 0.02, 2), and a weakly-informative prior *γ*_base_ ~ 𝒩(0.08, 0.03) centred on the cross-species mortality-rate-doubling-time–lifespan relationship recorded in the public AnAge database [13]. Posterior sampling used PyMC [36] NUTS (4 chains, 800 tune, 500 draws, target-accept 0.9); convergence was assessed by *R̂* (all = 1.000) and effective sample size. As a check robust to the non-differentiable matrix-exponential likelihood, the full phase-type first-passage model was also fitted by ensemble MCMC (emcee [37]; on the efficiency of non-local trial moves in Markov chain Monte Carlo, see [38]). The mean-field limit *E*[death age] = *α*(−*T*)^−1^**1** recovers a disposable-soma / reliability decomposition *τ*_eff_ ≈ *τ*_0_ *g*_maintenance_ *g*_allocation_ [12,39], of which the present model is the dynamical, distribution-level generalization. Robustness was examined against the number of axes, the number of load levels *L*, and the stationarity assumption.

### DNAm clock battery and latent localization

Epigenetic clocks were scored with a streaming pure-Python engine (clocks.py) implementing Horvath (with its anti-transform), Hannum, and PhenoAge, and, for reprogramming, the Ying et al. [18] DamAge/AdaptAge/CausAge causal clocks; mouse arrays used the universal mammalian clock [40]. EPICv2 probes were collapsed to base cg identifiers; clock CpGs missing from a matrix were mean-imputed from a reference panel (--impute-ref) and per-clock coverage reported. In HRS, protein-trained DNAm surrogate modules (inflammaging = DNAm CRP+GDF15+B2M; senescence = DNAm PAI-1+TIMP1; metabolic = DNAm HbA1c+leptin; renal = DNAm cystatin C) were added one at a time to the four blood axes (five-axis Bayesian generator models) and the reduction in latent fraction recorded; epigenetic ages, DunedinPACE and GrimAge were additionally Cox-modelled for mortality beyond age and blood axes. Whole-blood RNA-seq inflammaging/senescence gene modules (e.g. CDKN2A, CDKN1A, SERPINE1, GDF15, IL6, CXCL8, TNF, NFKB1) were tested in parallel by Cox regression.

### Mendelian randomization

Two-sample cis-pQTL Mendelian randomization [41] used genome-wide-significant (*p* < 5 × 10^−8^) variants within ±1 Mb of each gene from UKB-PPP (REGENIE summary statistics, GRCh38; effect allele = ALLELE1) and deCODE (SomaScan, GRCh38, with rsIDs). For UKB-PPP the cis lead SNP was mapped to an rsID via the Ensembl GRCh38 REST API; outcome effects came from the parental-lifespan GWAS (by rsID through the MRC IEU OpenGWAS API [42], or by GRCh38 position from the harmonised GWAS-Catalog file). Effects were harmonized to the protein-increasing allele (palindromic SNPs resolved or dropped by effect-allele frequency), and the Wald ratio (years of lifespan per SD protein) was computed with a delta-method standard error; where several independent instruments existed we used inverse-variance-weighted MR with MR-Egger and weighted-median sensitivity analyses. Exposures were the latent’s measurable components (GDF15, cystatin C/CST3, PAI-1/SERPINE1, TIMP1, B2M, IL6) and the growth-signalling/IIS axis (IGFBP1/2/3, GH1, GHR, IGF1R; IGF-1 from deCODE). **Known-causal positive controls (LPA, IL6R)** were analysed identically to calibrate power, and instrument strength was summarized by the F-statistic.

### Colocalization

For each cis region we ran coloc.abf [43], comparing the pQTL and lifespan GWAS over five hypotheses (H0 no association; H1/H2 one trait only; H3 distinct causal variants; H4 a shared causal variant). Per-SNP approximate Bayes factors used *z* = *β*/se and *V* = se^2^ with prior variance *W* = 0.15^2^ and priors *p*_1_ = *p*_2_ = 10^−4^, *p*_12_ = 10^−5^, over the ±100 kb cis window, matched to the lifespan GWAS by GRCh38 position (computed in log-space with the log-sum-exp identity). PP.H4 indexes colocalization and PP.H1 an exposure-only signal. The single-causal-variant assumption of coloc.abf is flagged at allelically heterogeneous loci such as LPA.

### Reprogramming reversibility

Processed EPIC/450K beta matrices were used where available; raw IDATs were processed to noob-normalized betas with methylprep [44] in an isolated Python 3.10 environment. Each dataset was scored with the chronological (Horvath, Hannum), phenotype (PhenoAge) and causality-enriched damage (DamAge/AdaptAge/CausAge, intercept-free, so only group differences are interpreted) clocks. Reversal was the reprogrammed − control difference, paired where an internal control existed (GSE165178: SSEA4^+^ reprogramming vs CD13^+^ non-reprogrammed cells; GSE142439: Treated vs Normal per donor). For the in-vivo mouse data (GSE190665, mammalian array, clock1 [40]) the treatment effect was age-adjusted by regression because treated animals were chronologically older. Processing was validated against authors’ reported DNAmAge (GSE142439 r = 0.97).

### Cross-species anchor and statistics

The Gompertz baseline prior was anchored by the cross-species mortality-rate-doubling-time–lifespan relationship in the public AnAge database [13] (s1_crossspecies_tau.py). Analyses used Python 3.12 (numpy, pandas, scipy, statsmodels), with isolated environments for PyMC/arviz/emcee (numpy < 2) and methylprep (Python 3.10, numpy < 2, pandas < 2), and base R 4.3 for .RDS/mammalian-array reading. Uncertainty is reported as 95% highest-density intervals (Bayesian) or 95% confidence intervals (frequentist). OpenGWAS and Synapse access tokens are read from local files and are never hard-coded.

### Data and code

All primary data are public or provider-restricted (NHANES + Linked Mortality; HRS [restricted; aggregate-only, local]; GEO; UKB-PPP via Synapse syn51365303; deCODE; GWAS Catalog GCST006697; AnAge). Full numerical results are provided as the S1–S11 Tables and code is archived at Zenodo (DOI: 10.5281/zenodo.20790403). HRS analyses are aggregate-only per data-use terms; restricted HRS individual-level data and licensed pQTL files are not redistributed.

## Supplementary Material

Supplement 1

Supplement 2

Supplement 3

Supplement 4

Supplement 5

Supplement 6

Supplement 7

Supplement 8

Supplement 9

Supplement 10

11

## Figures and Tables

**Table 1.** Generator-matrix decomposition of mortality acceleration (NHANES; replicated in HRS).

Quantity	Median [95% HDI]
Total Gompertz acceleration γ	0.094 [0.092, 0.097] /yr
Visible (measured-axis dynamics, Σβ_j_λ_j_)	0.0071 [0.0067, 0.0076]
Latent (γ_base)	0.087 [0.085, 0.090]
**Latent fraction (NHANES)**	**92.5% [92.0, 93.0]**
**Latent fraction (HRS replication)**	**91.6% [89.6, 93.4]**

Per-axis contribution (β·λ): A1 inflammation 0.00051; A2 metabolic 0.00197; A3 organ/renal 0.00144; A4 haematopoietic 0.00318. *Source:* bayes_unified_summary.csv, bayes_unified_axis.csv, HRS_bayes_generator.csv.

**Table 2.** cis-pQTL Mendelian randomization and colocalization of measurable aging-marker axes on human lifespan (Timmers 2019). MR = Wald-ratio (yr per SD protein); coloc = coloc.abf posterior of a shared causal variant (PP.H4) / exposure-only signal (PP.H1). Platforms: UKB-PPP (Olink), deCODE (SomaScan). Instrument strength = approximate F (UKB-PPP) or cis lead −log_10_P (deCODE/IIS). “—” = not testable (no usable cis-pQTL, or outcome-GWAS coverage gap).

Protein	Axis / role	Platform	Instrument	MR β (yr/SD)	p	coloc PP.H4	PP.H1
**LPA**	positive control	UKB-PPP	strong	+0.027	**9×10** ^**−12**^	(H3 = 1.00)†	0.00
**IL6R**	positive control	UKB-PPP	F=20031	−0.0086	**2.8×10** ^**−5**^	0.35	0.04
**IL6R**	positive control	deCODE	lead large	−0.0146	**3.8×10** ^**−6**^	0.36	0.04
GDF15	inflammaging (latent)	UKB-PPP	F=1924	+0.0033	0.68	0.001	0.36
GDF15	inflammaging (latent)	deCODE	strong	−0.0039	0.54	0.001	0.36
**CST3** (cystatin C)	renal (latent)	UKB-PPP	F=1499	+0.0017	0.84	0.002	**0.97**
**CST3** (cystatin C)	renal (latent)	deCODE	−log_10_P=1380	0.0017	0.87	0.002	**0.98**
SERPINE1 (PAI-1)	senescence (latent)	UKB-PPP	F=161	+0.055	0.022‡	0.03	0.96
B2M	inflammaging (latent)	UKB-PPP & deCODE	no cis-pQTL	—	—	—	—
IL6	inflammaging (latent)	UKB-PPP & deCODE	no cis-pQTL	—	—	—	—
TIMP1	senescence (latent)	UKB-PPP / deCODE	chrX / outcome gap	—	—	—	—
**IGFBP3** (IGF-1 carrier)	IIS/GH (hyperfunction)	UKB-PPP	F=837	−0.0013	0.79	0.00	**0.98**
IGFBP1	IIS/GH	UKB-PPP	lead 9.2	−0.083	0.093	0.00	0.74
IGF1R	IIS/GH	UKB-PPP	lead 48.5	+0.021	0.33	0.00	0.90
GHR	IIS/GH	UKB-PPP	lead 1398	+0.0087	0.018‡	0.02	0.97
GH1	IIS/GH	UKB-PPP	no cis-pQTL (lead 4.0)	—	—	—	—
IGFBP2	IIS/GH	UKB-PPP	outcome gap (chr2)	—	—	—	—
**IGF-1** (direct)	IIS/GH	deCODE	lead 11.8	(outcome gap at lead)	—	0.01	0.22

† LPA region has extreme allelic heterogeneity (KIV-2 CNV); coloc.abf (single-causal-variant) returns PP.H3≈1, a known limitation; the MR signal (p=9×10^−12^) stands.

‡ Nominal MR not supported by colocalization (PP.H4≤0.03) → attributable to LD. *Source:* MR_ukbppp.csv, MR_COLOC_decode.csv, MR_COLOC_iis.csv, MR_COLOC_igf1.csv, COLOC_ukbppp.csv. **Key:** positive controls detect causation; no measurable molecular component (inflammatory/renal, growth-signalling, epigenetic) shows a supported causal effect, and none colocalizes with lifespan.

**Table 3.** Reversibility: Δ epigenetic age (reprogrammed − control) for a chronological vs a causality-enriched damage clock, across reprogramming datasets. Δ in clock units (yr for age clocks; arbitrary units for DamAge, intercept-free → group difference only). Negative = reversal.

Clock	Type	MPTR (GSE165179)	Sendai (GSE165178)	mRNA (GSE142439)
**Horvath2013**	chronological	−**11.2**	−**22.1**	+0.15 §
PhenoAge	phenotype/mortality	−1.3	−14.1	+4.0
**DamAge**	causality-enriched damage (MR-derived)	**+13.2**	−2.2 (d9 +16.6)	+ 1.5
AdaptAge	causal-adaptive	−16.0	−16.9	−3.0

§ GSE142439 reversal is small and normalization-sensitive (authors’ DNAmAge −3.06; our noob processing +0.15; clock-vs-author r=0.97); an underpowered test of the dissociation. In-vivo mouse partial reprogramming (Browder, GSE190665): chronological mammalian clock −0.09 yr (age-adjusted, NS). *Source:* REV_damage.csv, REV_GSE165178.csv, REV_GSE142439.csv, TRACK2_mouse_clock_ageadj.csv. **Key:** the chronological clock reverses robustly across two reprogramming methods; the causality-enriched damage clock resists during identity-preserving (partial) reprogramming.

## Data Availability

All primary data are public (NHANES + Linked Mortality; HRS [restricted; analysed locally, aggregate-only]; GEO [GSE165178, GSE165179, GSE142439, GSE247179, GSE190665]; UKB-PPP via Synapse syn51365303; deCODE; GWAS Catalog GCST006697; AnAge). Analysis and figure code and all derived result tables are archived at Zenodo (DOI: 10.5281/zenodo.20790403) and provided as Supporting Information; restricted HRS individual-level data and licensed pQTL files (UKB-PPP, deCODE) are not redistributed and are obtained from their providers as described.
