# Supplementary material for: A generator-matrix causal-inference framework separates measurable aging biomarkers from mortality-driving latent dynamics in humans
Source: medRxiv. 2026 Jul 9:2026.07.05.26356402. Preprint. [Version 1] doi: 10.64898/2026.07.05.26356402 (PMC13370587; doi:10.64898/2026.07.05.26356402)
Supplement: Supplement 1 [file media-1.docx]

# STROBE-MR checklist — completed

**Manuscript:** A generator-matrix causal-inference framework separates measurable aging biomarkers from mortality-driving latent dynamics in humans (Tanigawa & Iwaki). Manuscript ID PCOMPBIOL-D-26-01514.

**Reference:** Skrivankova VW, Richmond RC, Woolf BAR, et al. Strengthening the Reporting of Observational Studies in Epidemiology Using Mendelian Randomization: The STROBE-MR Statement. *JAMA*. 2021;326(16):1614–1621.

*Scope note.* The Mendelian-randomization (MR) component of this study is one of three orthogonal analyses. It is a **two-sample, cis-pQTL MR with colocalization**, using publicly released GWAS/pQTL **summary statistics** (no individual-level genotype data). Page numbers refer to the submitted manuscript PDF (text + figures); where a sub-item is not applicable to summary-data cis-pQTL MR, this is stated.

| # | Section / topic — STROBE-MR recommendation | Reported (page) | Location and relevant text |
| --- | --- | --- | --- |
| 1 | **Title and abstract** — indicate MR as the study’s design in the title and/or abstract; give an informative, balanced summary | p1 | Title: “…cis-pQTL Mendelian randomization…” is described in the abstract. Abstract: “a positive-control-calibrated, two-platform cis-pQTL Mendelian-randomization and colocalization design (UKB-PPP, deCODE) against parental-lifespan GWAS tests whether the latent’s measurable components are causal.” |
| 2 | **Background/rationale** — scientific background; define exposure and outcome; state why a causal effect is plausible and why MR is informative | p2–3 (Introduction) | “Genetic causal tests of individual aging-associated proteins have been performed piecemeal and give mixed or null results, but rarely within a single framework that calibrates power with positive controls or guards against linkage-disequilibrium confounding by colocalization.” Exposure = circulating aging-biomarker proteins (inflammatory/renal, growth-signalling/IIS); outcome = human lifespan (parental lifespan). |
| 3 | **Objectives** — specific objectives and pre-specified causal hypotheses | p3 (Introduction) | “we test whether the measurable molecular components are **causal** for human lifespan using cis-pQTL Mendelian randomization plus colocalization on two independent proteomic platforms, anchored by known-causal positive controls so that a null cannot be dismissed as low power.” |
| 4 | **Study design** — key elements presented early; individual- vs summary-level data; one- vs two-sample | p1 abstract; p9–11 (Methods); Table 2 (p12–13) | Two-sample MR on **summary statistics**; two exposure platforms (UKB-PPP Olink; deCODE SomaScan) and a parental-lifespan outcome GWAS. Methods describe the cis-pQTL instrument selection and the coloc.abf design. |
| 5 | **Setting / data sources** — sources, settings and dates of the underlying GWAS | p9–11 (Methods); p21 (Data acknowledgements) | Exposure pQTL: UKB-PPP (Sun et al. 2023; Synapse syn51365303) and deCODE (Ferkingstad et al. 2021). Outcome: parental-lifespan GWAS (Timmers et al. 2019; GWAS Catalog GCST006697). |
| 6 | **Participants / populations** — eligibility and ancestry of the GWAS used (for two-sample MR, for both samples) | p8 (Limitations); p9–11; p21 | Exposure and outcome GWAS are predominantly **European-ancestry** population studies (UKB-PPP, deCODE, UK Biobank parental lifespan). External-validity restriction to European ancestry is acknowledged in Limitations. |
| 7 | **Variables** — clearly define exposures, outcome, confounders/positive controls | p9–11; Table 2 (p12–13) | Exposures: plasma protein levels (cis-pQTL-instrumented): LPA, IL6R (positive controls); GDF15, CST3/cystatin C, SERPINE1, B2M, IL6, TIMP1; IGFBP3, IGFBP1, IGF1R, GHR, GH1, IGFBP2, IGF-1. Outcome: lifespan. |
| 8 | **Instruments** — how genetic variants were chosen; instrument strength | p11 (Methods); Table 2 | cis-pQTL lead variants within the protein-coding gene region; instrument strength reported as approximate F-statistic (UKB-PPP) or cis-lead −log₁₀P (deCODE/IIS). “Instruments were strong where proteins could be instrumented (F = 1499–20031)” (Results p5). |
| 9 | **Sources of bias** — pleiotropy, LD confounding, population stratification, etc. | p5 (Results); p11 (Methods); p8 (Limitations) | **cis-only** instruments limit horizontal pleiotropy; **colocalization (coloc.abf)** distinguishes a shared causal variant (PP.H4) from LD-confounded co-location (PP.H1). “The single nominal signal (SERPINE1, p=0.022) did not colocalize (PP.H4 = 0.03) and is attributable to LD.” |
| 10 | **Statistical methods** — causal estimator, sensitivity analyses, software/packages | p11 (Methods) | Wald-ratio MR (yr per SD protein); colocalization via coloc.abf (posterior PP.H0–H4). Two independent platforms and three longevity outcomes as sensitivity/replication. Software/packages and versions are archived with the analysis code (Zenodo; environment/). |
| 11 | **Assessment of assumptions** — relevance, independence, exclusion restriction | p5 (Results); p11 (Methods) | **Relevance:** F-statistics (1499–20031) and strong cis leads. **Exclusion restriction:** cis-pQTL + colocalization (PP.H4) guard against pleiotropy/LD. **Power/validity calibration:** known-causal **positive controls (LPA, IL6R)** are detected, so a null is not attributable to low power. |
| 12 | **Descriptive data** — instrument and GWAS characteristics | Table 2 (p12–13) | Per-protein platform, instrument strength (F / cis-lead −log₁₀P), MR β (yr/SD), p, coloc PP.H4 and PP.H1; “—” entries flag proteins not testable (no usable cis-pQTL; or outcome-GWAS coverage gap). |
| 13 | **Main results** — causal estimates with confidence intervals | p5 (Results); Table 2 | Positive controls: LPA β=+0.027 [95% CI 0.019, 0.035] yr/SD, p=9×10⁻¹²; IL6R β=−0.0086 [−0.013, −0.0047], p=2.8×10⁻⁵. Latent components null: cystatin C β=+0.0017 [−0.014, 0.018]; GDF15 +0.0033 [−0.013, 0.019]; IGFBP3 null with PP.H4=0. |
| 14 | **Other analyses** — sensitivity, replication, colocalization | p5 (Results); Table 2 | Cross-platform replication (UKB-PPP and deCODE) and three longevity outcomes; colocalization for every testable protein. “Results were unchanged across the two MR/colocalization implementations and the three longevity outcomes tested.” |
| 15 | **Key results** — summarize with reference to objectives | p6 (Discussion) | “the measurable inflammatory/senescence/renal proxies are markers not causes” and “the measurable circulating growth-signalling axis joins the inflammatory/renal axis as a marker, not a cause, of human longevity.” |
| 16 | **Limitations** — IV assumptions, direction/magnitude of bias, instrument gaps | p8 (Limitations) | “MR is constrained by instrument availability: B2M and IL6 lack usable cis-pQTL on both platforms, TIMP1 and IGFBP2 fall in outcome-GWAS gaps (chromosome X; a chr2 coverage gap), and coloc.abf assumes a single causal variant per region (limiting interpretation at allelically heterogeneous loci such as LPA). Circulating pQTL-MR indexes secreted proteins, not intracellular signalling (mTOR)…” |
| 17 | **Interpretation** — cautious overall interpretation; mechanism | p6–8 (Discussion) | The measurable proxies index mortality risk, not mechanism; “this does not refute the hyperfunction mechanism but shows it has no causal handle in the measurable circulating proxy.” Interpretation is triangulated with the generator-matrix and reprogramming analyses. |
| 18 | **Generalizability** — external validity | p8 (Limitations) | Results derive from predominantly European-ancestry GWAS and index the **circulating** proteome; generalization beyond European-ancestry populations and to intracellular signalling is explicitly bounded. |
| 19 | **Funding** — sources and role of funders | p21 (Declarations) | “This work was supported by JSPS KAKENHI Grant Number JP24K15812 (to M.T.).” The funders had no role in study design, analysis, decision to publish, or preparation of the manuscript. |
| 20 | **Data and code availability** — data, code, and scripts to replicate | p21 (Declarations) | Analysis/figure code and derived result tables: Zenodo DOI 10.5281/zenodo.20790403. Exposure pQTL: UKB-PPP (Synapse syn51365303), deCODE; outcome: GWAS Catalog GCST006697. Licensed pQTL files are obtained from the providers (not redistributed). |

*Completed by the authors. Page numbers refer to the submitted manuscript PDF (combined text and figures).*
