# Supplementary material for: A generator-matrix causal-inference framework separates measurable aging biomarkers from mortality-driving latent dynamics in humans
Source: medRxiv. 2026 Jul 9:2026.07.05.26356402. Preprint. [Version 1] doi: 10.64898/2026.07.05.26356402 (PMC13370587; doi:10.64898/2026.07.05.26356402)
Supplement: 11 [file NIHPP2026.07.05.26356402V1-supplement-1.pdf]

medRxiv preprint doi: https://doi.org/10.64898/2026.07.05.26356402; this version posted July 9, 2026. The copyright holder for this preprint (which was not certified by peer review) is the author/funder, who has granted medRxiv a license to display the preprint in perpetuity.
It is made available under a CC-BY 4.0 International license .

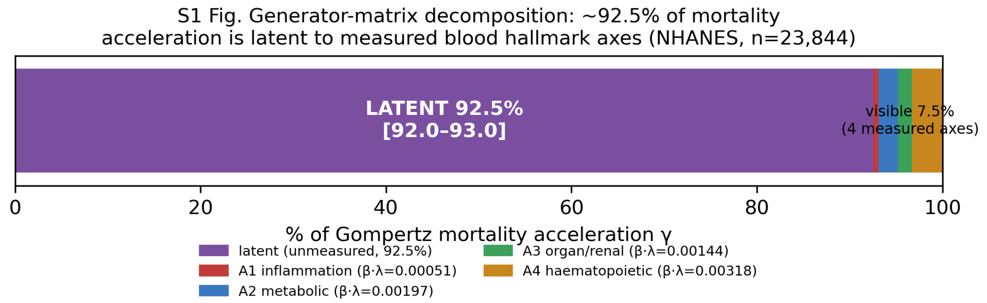

S1 Fig. Generator-matrix decomposition of mortality acceleration (`fig_latent_decomp.png`): 92.5% latent vs 7.5% visible (4 measured axes with β·λ contributions), NHANES.

medRxiv preprint doi: https://doi.org/10.64898/2026.07.05.26356402; this version posted July 9, 2026. The copyright holder for this preprint (which was not certified by peer review) is the author/funder, who has granted medRxiv a license to display the preprint in perpetuity.
It is made available under a CC-BY 4.0 International license .

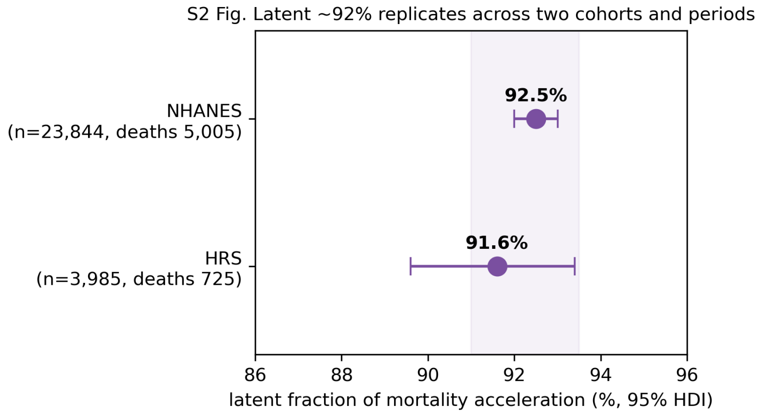

S2 Fig. Two-cohort replication of the latent fraction (`fig_cohort_replication.png`): NHANES 92.5% / HRS 91.6%.

medRxiv preprint doi: https://doi.org/10.64898/2026.07.05.26356402; this version posted July 9, 2026. The copyright holder for this preprint (which was not certified by peer review) is the author/funder, who has granted medRxiv a license to display the preprint in perpetuity.
It is made available under a CC-BY 4.0 International license .

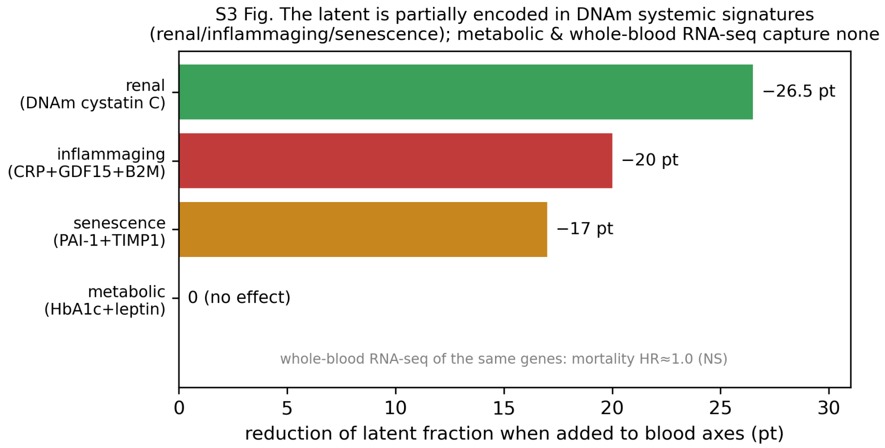

S3 Fig. DNAm systemic-signature decomposition of the latent (`fig_dnam_modules.png`): renal −26.5 / inflammaging −20 / senescence −17 / metabolic 0 pt; whole-blood RNA-seq NS.

Further supplementary: per-dataset reversibility (`REV_*`); cross-species τ anchor (`s1_crossspecies_tau`).

---

medRxiv preprint doi: https://doi.org/10.64898/2026.07.05.26356402; this version posted July 9, 2026. The copyright holder for this preprint (which was not certified by peer review) is the author/funder, who has granted medRxiv a license to display the preprint in perpetuity.
It is made available under a CC-BY 4.0 International license .

Supporting information

All Supporting Information files are derived or aggregate results computed in this study; no individual-level or restricted primary data are included. HRS-derived tables are aggregate (cell sizes ≥ 5) under the HRS data-use terms. Primary data are obtained from the sources cited under Data and code availability.

S1 Fig. Generator-matrix decomposition of mortality acceleration. 92.5% latent versus 7.5% visible (four measured axes with β·λ contributions). *Source:* NHANES 1999–2010 + Linked Mortality. (`fig_latent_decomp`; `S1_Fig`)

S2 Fig. Two-cohort replication of the latent fraction. NHANES 92.5% / HRS 91.6%. *Source:* NHANES + HRS (aggregate). (`fig_cohort_replication`; `S2_Fig`)

S3 Fig. DNAm systemic-signature decomposition of the latent. Renal −26.5 / inflammaging −20 / senescence −17 / metabolic 0 pt; whole-blood RNA-seq not significant. *Source:* HRS (aggregate). (`fig_dnam_modules`; `S3_Fig`)

S1 Table. Generator-matrix posterior (unified model). Posterior summaries for

medRxiv preprint doi: https://doi.org/10.64898/2026.07.05.26356402; this version posted July 9, 2026. The copyright holder for this preprint (which was not certified by peer review) is the author/funder, who has granted medRxiv a license to display the preprint in perpetuity.
It is made available under a CC-BY 4.0 International license .

the joint biomarker-and-mortality model. *Source:* NHANES 1999–2010 + Linked Mortality (n = 23,844). (`bayes_unified_summary.csv`, `bayes_unified_axis.csv`)

S2 Table. HRS replication and blood-axis mediation. Latent-fraction replication and Cox mediation by DNAm modules. *Source:* HRS (aggregate; n = 3,985, cell sizes ≥ 5). (`HRS_bayes_generator.csv`, `HRS_dnam_cox.csv`)

S3 Table. DNAm module decomposition of the latent. Reduction in latent fraction on adding each protein-trained DNAm module. *Source:* HRS (aggregate; n = 3,985, cell sizes ≥ 5). (`HRS_dnam_modules.csv`)

S4 Table. Whole-blood RNA-seq module mortality hazard ratios. *Source:* HRS (aggregate; n = 3,651, cell sizes ≥ 5). (`HRS_rnaseq_modules.csv`)

S5 Table. Mendelian randomization, UKB-PPP. Wald-ratio MR for latent components and known-causal positive controls. *Source:* UKB-PPP pQTL × parental-lifespan GWAS (GCST006697). (`MR_ukbppp.csv`)

S6 Table. Mendelian randomization and colocalization, deCODE. Cross-platform replication. *Source:* deCODE pQTL × parental-lifespan GWAS. (`MR_COLOC_decode.csv`)

S7 Table. Mendelian randomization and colocalization, growth-signalling/IIS axis. *Source:* UKB-PPP and deCODE pQTL × parental-lifespan GWAS. (`MR_COLOC_iis.csv`, `MR_COLOC_igf1.csv`)

S8 Table. Colocalization, UKB-PPP. Per-region coloc.abf posterior probabilities. *Source:* UKB-PPP pQTL × parental-lifespan GWAS. (`COLOC_ukbppp.csv`)

S9 Table. Reprogramming reversibility, per-dataset clock outputs. *Source:* GEO GSE165179 (MPTR), GSE165178 (Sendai), GSE142439 (mRNA); public in-vitro reprogramming datasets. (`REV_damage.csv`, `REV_GSE165178.csv`, `REV_GSE142439.csv`)

S10 Table. In-vivo mouse reprogramming (age-adjusted). *Source:* GEO GSE190665 (public). (`TRACK2_mouse_clock_ageadj.csv`)

S11 Table. Cross-species mortality-rate-doubling-time–lifespan anchor. *Source:* AnAge database (public). (`S1_crossspecies_scaling.csv`)
